# 3-[1-(4-Bromo­phen­yl)eth­oxy]-2,2,5-trimethyl-4-phenyl-3-aza­hexa­ne

**DOI:** 10.1107/S1600536813029966

**Published:** 2013-11-20

**Authors:** Praveen Pitliya, Ray J. Butcher, A. Karim, Paul F. Hudrlik, Anne M. Hudrlik, D. Raghavan

**Affiliations:** a Department of Chemistry, Howard University, 525 College Street NW, Washington, DC 20059, USA; b Department of Polymer Engineering, University of Akron, Akron, OH, USA

## Abstract

The title compound, C_22_H_30_BrNO, is an alk­oxy­amine compound, an effective initiator in nitroxide-mediated free radical polymerization. It was prepared as a mixture of two diasteromers; the crystal for the X-ray analysis showed one of these as a pair of *R*,*S* and *S*,*R* enanti­omers. The *tert*-butyl and isopropyl groups are in an almost *anti* conformation in the crystal [C—N—C—C torsion angle = −168.8 (1)°], and the methyl group of the ethoxy group is in an approximate *anti* relationship to the *tert*-butyl group. The dihedral angle between the phenyl and benzene rings is 33.12 (7)°. The Br atom is disordered over two positions, with occupancies of 0.9139 (16) and 0.0861 (16). In the crystal, weak C—H⋯Br contacts link the mol­ecules into chains along [-110].

## Related literature

For the use of TIPNO-based alk­oxy­amine in polymer synthesis (TIPNO = 2,2,5-trimethyl-4-phenyl-3-aza­hexane-3-nitroxide), see: Benoit *et al.* (1999 ▶). For the synthesis of the title compound, see: Kaul *et al.* (2010 ▶). For the use of the title compound in block copolymer synthesis, see: Richard *et al.* (2008 ▶); Kaul *et al.* (2010 ▶); Stalmach *et al.* (2001 ▶); van der Veen *et al.* (2004 ▶); Widin *et al.* (2013 ▶). For properties of alk­oxy­amines, see: Benoit *et al.* (1999 ▶); Wetter *et al.* (2004 ▶); Rodlert *et al.* (2000 ▶); Nilsen & Braslau (2006 ▶). For synthesis of 1-(4-bromo­phen­yl) ethyl­bromide, see: Kodama *et al.* (2011 ▶); Thompson *et al.* (2011 ▶). For standard bond lengths, see: Allen *et al.* (1987 ▶).
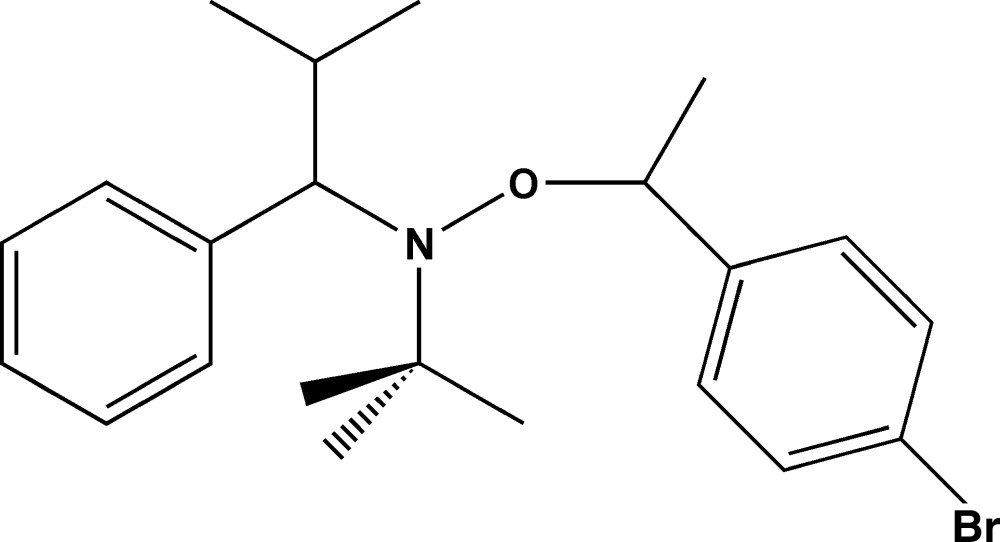


## Experimental

### 

#### Crystal data

C_22_H_30_BrNO
*M* *_r_* = 404.38
Triclinic, 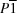

*a* = 8.2595 (4) Å
*b* = 10.0175 (4) Å
*c* = 12.5257 (6) Å
α = 88.798 (4)°
β = 78.824 (4)°
γ = 89.220 (3)°
*V* = 1016.45 (8) Å^3^
*Z* = 2
Mo *K*α radiation
μ = 2.03 mm^−1^
*T* = 123 K
0.51 × 0.35 × 0.06 mm

#### Data collection

Agilent Xcalibur (Ruby, Gemini) diffractometer
Absorption correction: analytical [*CrysAlis PRO* (Agilent, 2012 ▶), based on expressions derived by Clark & Reid (1995 ▶)] *T*_min_ = 0.375, *T*_max_ = 0.888
23820 measured reflections
13014 independent reflections
7448 reflections with *I* > 2σ(*I*)
*R*_int_ = 0.040

#### Refinement

*R*[*F* ^2^ > 2σ(*F* ^2^)] = 0.066
*wR*(*F* ^2^) = 0.123
*S* = 1.08
13014 reflections
237 parameters
14 restraints
H-atom parameters constrained
Δρ_max_ = 0.71 e Å^−3^
Δρ_min_ = −0.78 e Å^−3^

### 

Data collection: *CrysAlis PRO* (Agilent, 2012 ▶); cell refinement: *CrysAlis PRO*; data reduction: *CrysAlis PRO*; program(s) used to solve structure: *SHELXS97* (Sheldrick, 2008 ▶); program(s) used to refine structure: *SHELXL97* (Sheldrick, 2008 ▶); molecular graphics: *SHELXTL* (Sheldrick, 2008 ▶); software used to prepare material for publication: *SHELXTL*.

## Supplementary Material

Crystal structure: contains datablock(s) I, New_Global_Publ_Block. DOI: 10.1107/S1600536813029966/hg5350sup1.cif

Structure factors: contains datablock(s) I. DOI: 10.1107/S1600536813029966/hg5350Isup2.hkl

Supplementary material file. DOI: 10.1107/S1600536813029966/hg5350Isup3.cml

Additional supplementary materials:  crystallographic information; 3D view; checkCIF report

## Figures and Tables

**Table 1:** Hydrogen-bond geometry (Å, °)

*D*—H⋯*A*	*D*—H	H⋯*A*	*D*⋯*A*	*D*—H⋯*A*
C11—H11*B*⋯Br1*A* ^i^	0.98	3.12	3.9782 (16)	148
C13—H13*A*⋯Br1*A* ^ii^	1.00	3.07	4.0358 (13)	163
C13—H13*A*⋯Br1*B* ^ii^	1.00	3.02	3.970 (2)	159
C15—H15*B*⋯Br1*B* ^ii^	0.98	3.14	4.017 (3)	149

Symmetry codes: (i) 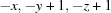; (ii) 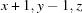.

**Fig. 1.**
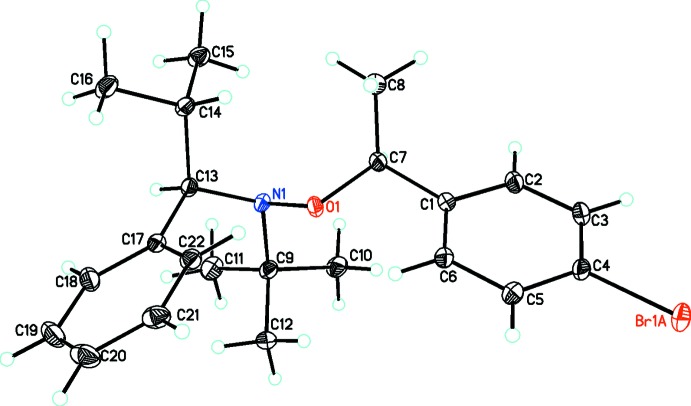
Structural diagram of the title compound. Atomic displacement parameters are at the 30% probability level.

**Fig. 2.**
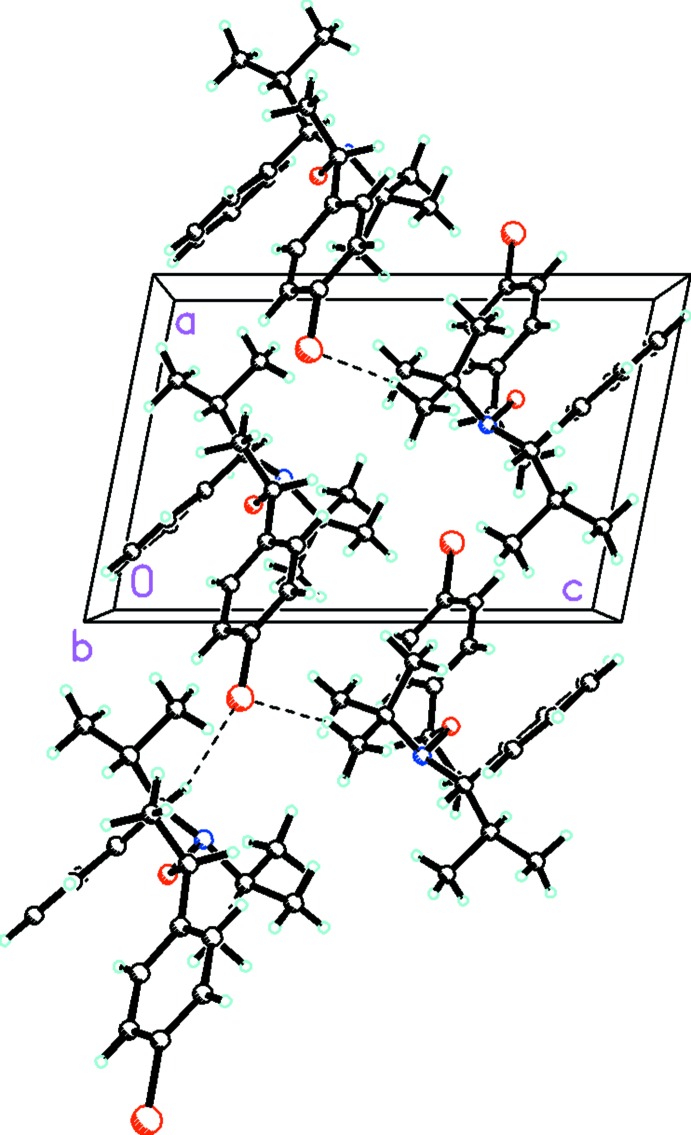
Packing diagram of the tile compound viewed along the a axis.

**Fig. 3.**
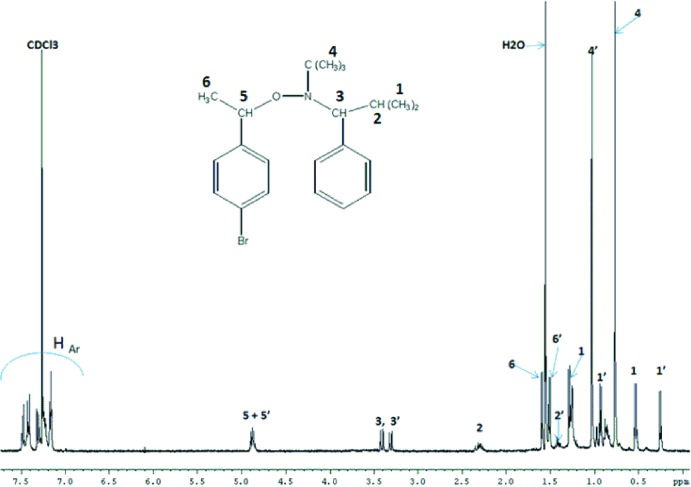
1H NMR spectrum of 2,2,5-trimethyl-(1'-p-bromophenylethoxy)-4-phenyl-3-azahexane (two diastereomers) in CDCl3

## supplementary crystallographic information

### 1. Comment

Nitroxide-mediated radical polymerization (NMRP) has been widely used for polymer and block copolymer synthesis, with controlled molecular weight, polydispersity, and end group functionality, and with relatively mild conditions (Benoit *et al.*, 1999; Wetter *et al.*, 2004). In NMRP, an alkoxyamine is used to initiate and mediate polymerization. The C–O bond of an alkoxyamine is stable to normal purification and handling procedures as well as variety of reaction conditions (Rodlert *et al.*, 2000). However, under appropriate conditions, homolysis of the C—O bond occurs, giving a (relatively stable) nitroxide radical and carbon radical which can initiate radical polymerization. The nitroxides are kinetically persistent radicals, and act as reversible traps for the less stable radical intermediates in the polymerization (Nilsen & Braslau, 2006).

Alkoxyamines based on 2,2,5-trimethyl-4-phenyl-3-azahexane-3-nitroxide (TIPNO) have been widely used as initiators/mediators to synthesize a variety of linear polymers and block copolymers (van der Veen *et al.*, 2004; Widin *et al.*, 2013). In particular, alkoxyamine 2 (*X* = H), was found to be especially useful in NMRP (Benoit *et al.*, 1999). The title compound, bromo analog 1 (*X* = Br) was developed so that 2 could be attached to a polymer, to give a macroinitiator for block copolymer synthesis (Stalmach *et al.*, 2001). It has been used successfully in a number of block copolymer synthesis including polystyrene-b-poly (3-hexyl thiophene) (PS—P3HT) (Kaul *et al.*, 2010), poly (phenylene vinylene)-b-poly (butylacrylate) (PPV-b-PBA)(Stalmach *et al.*, 2001), and poly(3-hexylthiophene)-b-poly(butylacrylate-stat- chloromethylstyrene)-C60 [P(BA-stat-CMS)]-C60 (Richard *et al.*, 2008).

The title compound was prepared as a mixture of two diastereomers in nearly equal amounts, as can be seen by the NMR spectrum (Figure 3). The crystal for the X-ray analysis showed one of the diastereomers, a pair of *R*,*S* and S,*R* (at C7 and C13) enantiomers. The *tert*-butyl group and isopropyl group are in a nearly *anti* conformation in the crystal (dihedral angle C9, N1, C13, C14 = -168.8 (1)°). The C—Me bond (C7, C8) and N-*tert*-Bu bond (N1, C9) are in an *anti* relationship (C8, C7, N1, C9 = 172.3 (1)°), and the C—Me bond (C7, C8) and *C*-isopropyl bond (C13, C14) are in a *syn* relationship (C8, C7, C13, C14 = 4.6 (1) degrees). The dihedral angle between the planes of the phenyl groups is 33.12 (7)°.

The bromine atom was disordered over two positions with occupancies of 0.9139 (16) and 0.0961 (16). The central N is pyramidal and all the bond lengths and angles are in the normal ranges (Allen *et al.*, 1987). There are weak C—H···Br intermolecular contacts which link the molecules into chains in the [-1 1 0] direction.

### 2. Experimental

(*a*) 1-(4-Bromophenyl)ethylbromide

1-(4-Bromophenyl)ethylbromide was synthesized according to previously reported procedures. 4-Bromoacetophenone was reduced to 1-(4-bromophenyl)ethanol by sodium borohydride (Kodama *et al.*, 2011), and the product was treated with PBr_3_ using a procedure of Thompson (Thompson *et al.*, 2011). 1-(4-Bromophenyl)ethylbromide (Kaul *et al.*, 2010) was obtained as colorless liquid in 70% overall yield.

(*b*) 2,2,5-Trimethyl-(1'-*p*-bromophenylethoxy)-4-phenyl-3-αzahexane (1)

2,2,5-Trimethyl-(1'-*p*-bromophenylethoxy)-4-phenyl-3-azahexane was synthesized according to a previously reported method [Kaul *et al.*, 2010] with a modification of the time and temperature. Synthesized 1-(4-bromophenyl) ethylbromide and commercially available 2,2,5-trimethyl-4-phenyl-3-azahexane-3-nitroxide (TIPNO) were reacted in the presence of CuBr and *N*,*N*,*N*'\',*N*',*N*''-pentamethyldiethylenetriamine (PMDETA) in toluene at 75 *^o^*C for 17 hr to form the title compound (1), 2,2,5-trimethyl-(1'-*p*-bromophenylethoxy)-4-phenyl-3-azahexane, in 80% yield as a viscous colorless oil which slowly crystallized in the refrigerator. The synthesized compound was characterized by ^1^H NMR and its crystal structure was determined by X-ray crystallography. The ^1^H NMR spectrum (Figure 3) showed the presence of two diastereomers in nearly equal amounts, and was in agreement with the published spectrum (Kaul *et al.*, 2010). A crystal was taken for X-ray crystallography.

^1^H NMR (400 MHz, CDCl3) (mixture of two diastereoisomeres): δ (p.p.m.) = 7.55–7.15 (HAr), 4.89 (H5 and H5'), 3.43 (H3), 3.31 (H3'), 2.31 (H2), 1.58 (H6), 1.53 (H6'), 1.42 (H2'), 1.26 (H1), 1.04 (H4'), 0.93 (H1'), 0.78 (H4), 0.54 (H1), 0.25 (H1').

### 3. Refinement

Carbon-bonded hydrogen atoms were included in idealized positions and set to ride on the parent atoms. The bromine atom was disordered over two positions with occupancies of 0.9139 (16) and 0.0961 (16).

### Crystal data

**Table:**

C_22_H_30_BrNO	*Z* = 2
*M**_r_* = 404.38	*F*(000) = 424
Triclinic, *P*1	*D*_x_ = 1.321 Mg m^−^^3^
Hall symbol: -P 1	Mo *K*α radiation, λ = 0.71073 Å
*a* = 8.2595 (4) Å	Cell parameters from 4369 reflections
*b* = 10.0175 (4) Å	θ = 3.2–40.9°
*c* = 12.5257 (6) Å	µ = 2.03 mm^−^^1^
α = 88.798 (4)°	*T* = 123 K
β = 78.824 (4)°	Diamond shaped plate, colorless
γ = 89.220 (3)°	0.51 × 0.35 × 0.06 mm
*V* = 1016.45 (8) Å^3^	

### Data collection

**Table:**

Agilent Xcalibur (Ruby, Gemini) diffractometer	13014 independent reflections
Radiation source: Enhance (Mo) X-ray Source	7448 reflections with *I* > 2σ(*I*)
Graphite monochromator	*R*_int_ = 0.040
Detector resolution: 10.5081 pixels mm^-1^	θ_max_ = 41.0°, θ_min_ = 3.2°
ω scans	*h* = −13→15
Absorption correction: analytical [*CrysAlis PRO* (Agilent, 2012), based on expressions derived by Clark & Reid (1995)]	*k* = −18→18
*T*_min_ = 0.375, *T*_max_ = 0.888	*l* = −16→22
23820 measured reflections	

### Refinement

**Table:**

Refinement on *F*^2^	Primary atom site location: structure-invariant direct methods
Least-squares matrix: full	Secondary atom site location: difference Fourier map
*R*[*F*^2^ > 2σ(*F*^2^)] = 0.066	Hydrogen site location: inferred from neighbouring sites
*wR*(*F*^2^) = 0.123	H-atom parameters constrained
*S* = 1.08	*w* = 1/[σ^2^(*F*_o_^2^) + (0.0319*P*)^2^ + 0.3044*P*] where *P* = (*F*_o_^2^ + 2*F*_c_^2^)/3
13014 reflections	(Δ/σ)_max_ = 0.002
237 parameters	Δρ_max_ = 0.71 e Å^−^^3^
14 restraints	Δρ_min_ = −0.78 e Å^−^^3^

### Special details

**Table:**

Experimental. CrysAlisPro (Agilent, 2012) Analytical numeric absorption correction using a multifaceted crystal model based on expressions derived by R.C. Clark & J.S. Reid. (Clark & Reid, 1995)
Geometry. All e.s.d.'s (except the e.s.d. in the dihedral angle between two l.s. planes) are estimated using the full covariance matrix. The cell e.s.d.'s are taken into account individually in the estimation of e.s.d.'s in distances, angles and torsion angles; correlations between e.s.d.'s in cell parameters are only used when they are defined by crystal symmetry. An approximate (isotropic) treatment of cell e.s.d.'s is used for estimating e.s.d.'s involving l.s. planes.
Refinement. Refinement of *F*^2^ against ALL reflections. The weighted *R*-factor *wR* and goodness of fit *S* are based on *F*^2^, conventional *R*-factors *R* are based on *F*, with *F* set to zero for negative *F*^2^. The threshold expression of *F*^2^ > σ(*F*^2^) is used only for calculating *R*-factors(gt) *etc*. and is not relevant to the choice of reflections for refinement. *R*-factors based on *F*^2^ are statistically about twice as large as those based on *F*, and *R*- factors based on ALL data will be even larger.

### Fractional atomic coordinates and isotropic or equivalent isotropic displacement parameters (Å^2^)

**Table:**

	*x*	*y*	*z*	*U*_iso_*/*U*_eq_	Occ. (<1)
Br1A	−0.21657 (3)	1.03569 (3)	0.31920 (3)	0.03904 (8)	0.9139 (16)
Br1B	−0.2097 (3)	1.0344 (3)	0.2954 (4)	0.03904 (8)	0.0861 (16)
O1	0.33888 (11)	0.52249 (9)	0.26150 (7)	0.02013 (18)	
N1	0.42054 (13)	0.41412 (11)	0.31003 (9)	0.0184 (2)	
C1	0.22930 (16)	0.73932 (14)	0.30589 (11)	0.0213 (2)	
C2	0.21935 (18)	0.85614 (15)	0.36510 (12)	0.0263 (3)	
H2A	0.3043	0.8757	0.4036	0.032*	
C3	0.08780 (19)	0.94442 (15)	0.36896 (13)	0.0282 (3)	
H3A	0.0825	1.0244	0.4089	0.034*	
C4	−0.03621 (15)	0.91347 (13)	0.31314 (12)	0.0255 (3)	
C5	−0.03140 (18)	0.79814 (15)	0.25483 (12)	0.0260 (3)	
H5A	−0.1177	0.7784	0.2175	0.031*	
C6	0.10213 (17)	0.71062 (15)	0.25143 (11)	0.0235 (3)	
H6A	0.1066	0.6306	0.2116	0.028*	
C7	0.38199 (16)	0.65120 (14)	0.29630 (12)	0.0243 (3)	
H7A	0.4127	0.6411	0.3695	0.029*	
C8	0.5227 (2)	0.71609 (18)	0.21809 (15)	0.0361 (4)	
H8A	0.6227	0.6610	0.2138	0.054*	
H8B	0.5424	0.8050	0.2440	0.054*	
H8C	0.4943	0.7243	0.1458	0.054*	
C9	0.29326 (17)	0.35139 (15)	0.39863 (11)	0.0228 (3)	
C10	0.25278 (19)	0.45110 (17)	0.49164 (12)	0.0297 (3)	
H10A	0.1974	0.5298	0.4670	0.045*	
H10B	0.3551	0.4782	0.5133	0.045*	
H10C	0.1800	0.4089	0.5539	0.045*	
C11	0.3726 (2)	0.22698 (17)	0.44159 (13)	0.0338 (4)	
H11A	0.3849	0.1568	0.3872	0.051*	
H11B	0.3021	0.1950	0.5092	0.051*	
H11C	0.4813	0.2497	0.4559	0.051*	
C12	0.13032 (17)	0.31498 (16)	0.36504 (13)	0.0276 (3)	
H12A	0.1510	0.2450	0.3104	0.041*	
H12B	0.0841	0.3942	0.3342	0.041*	
H12C	0.0520	0.2825	0.4290	0.041*	
C13	0.49844 (15)	0.32510 (13)	0.21998 (10)	0.0188 (2)	
H13A	0.5453	0.2479	0.2563	0.023*	
C14	0.64814 (15)	0.39403 (15)	0.14855 (10)	0.0209 (2)	
H14A	0.6102	0.4801	0.1192	0.025*	
C15	0.77943 (17)	0.42426 (17)	0.21525 (12)	0.0273 (3)	
H15A	0.8661	0.4789	0.1713	0.041*	
H15B	0.8277	0.3404	0.2367	0.041*	
H15C	0.7287	0.4729	0.2805	0.041*	
C16	0.72633 (18)	0.30662 (18)	0.05259 (12)	0.0303 (3)	
H16A	0.8242	0.3510	0.0108	0.045*	
H16B	0.6463	0.2931	0.0055	0.045*	
H16C	0.7585	0.2200	0.0802	0.045*	
C17	0.38509 (16)	0.26336 (14)	0.15192 (10)	0.0203 (2)	
C18	0.3761 (2)	0.12507 (16)	0.14801 (12)	0.0283 (3)	
H18A	0.4397	0.0712	0.1881	0.034*	
C19	0.2754 (2)	0.06402 (18)	0.08627 (13)	0.0363 (4)	
H19A	0.2704	−0.0306	0.0847	0.044*	
C20	0.1827 (2)	0.1417 (2)	0.02737 (13)	0.0372 (4)	
H20A	0.1125	0.1007	−0.0138	0.045*	
C21	0.19305 (18)	0.27933 (18)	0.02873 (12)	0.0299 (3)	
H21A	0.1304	0.3327	−0.0124	0.036*	
C22	0.29426 (17)	0.34049 (15)	0.08968 (11)	0.0232 (3)	
H22A	0.3016	0.4351	0.0890	0.028*	

### Atomic displacement parameters (Å^2^)

**Table:**

	*U*^11^	*U*^22^	*U*^33^	*U*^12^	*U*^13^	*U*^23^
Br1A	0.03220 (8)	0.02458 (8)	0.0589 (2)	0.01144 (6)	−0.00651 (9)	0.00285 (9)
Br1B	0.03220 (8)	0.02458 (8)	0.0589 (2)	0.01144 (6)	−0.00651 (9)	0.00285 (9)
O1	0.0218 (4)	0.0146 (4)	0.0256 (4)	0.0023 (3)	−0.0084 (3)	−0.0031 (3)
N1	0.0176 (4)	0.0182 (5)	0.0187 (5)	0.0036 (4)	−0.0019 (4)	−0.0007 (4)
C1	0.0193 (5)	0.0165 (5)	0.0273 (6)	−0.0007 (4)	−0.0026 (5)	−0.0019 (5)
C2	0.0260 (6)	0.0197 (6)	0.0342 (7)	0.0003 (5)	−0.0079 (5)	−0.0059 (5)
C3	0.0305 (7)	0.0169 (6)	0.0357 (7)	0.0018 (5)	−0.0020 (6)	−0.0042 (5)
C4	0.0219 (6)	0.0173 (6)	0.0346 (7)	0.0035 (5)	0.0004 (5)	0.0046 (5)
C5	0.0217 (6)	0.0256 (7)	0.0310 (7)	0.0031 (5)	−0.0061 (5)	−0.0009 (5)
C6	0.0204 (5)	0.0207 (6)	0.0295 (6)	0.0011 (5)	−0.0043 (5)	−0.0060 (5)
C7	0.0187 (5)	0.0182 (6)	0.0367 (7)	−0.0001 (5)	−0.0067 (5)	−0.0058 (5)
C8	0.0243 (7)	0.0267 (8)	0.0543 (10)	−0.0028 (6)	0.0009 (7)	−0.0064 (7)
C9	0.0204 (5)	0.0230 (6)	0.0229 (6)	0.0009 (5)	0.0011 (5)	0.0002 (5)
C10	0.0278 (7)	0.0352 (8)	0.0232 (6)	−0.0001 (6)	0.0025 (5)	−0.0050 (6)
C11	0.0355 (8)	0.0317 (8)	0.0307 (7)	0.0048 (7)	0.0008 (6)	0.0089 (6)
C12	0.0206 (6)	0.0255 (7)	0.0342 (7)	−0.0038 (5)	0.0018 (5)	−0.0030 (6)
C13	0.0181 (5)	0.0182 (5)	0.0200 (5)	0.0024 (4)	−0.0036 (4)	−0.0023 (4)
C14	0.0165 (5)	0.0248 (6)	0.0208 (6)	0.0017 (5)	−0.0024 (4)	−0.0032 (5)
C15	0.0188 (5)	0.0360 (8)	0.0281 (7)	0.0005 (5)	−0.0060 (5)	−0.0057 (6)
C16	0.0221 (6)	0.0413 (9)	0.0260 (7)	0.0020 (6)	−0.0001 (5)	−0.0110 (6)
C17	0.0179 (5)	0.0213 (6)	0.0209 (6)	−0.0003 (5)	−0.0012 (4)	−0.0039 (4)
C18	0.0334 (7)	0.0221 (6)	0.0289 (7)	−0.0010 (6)	−0.0047 (6)	−0.0042 (5)
C19	0.0455 (9)	0.0276 (8)	0.0352 (8)	−0.0129 (7)	−0.0048 (7)	−0.0080 (6)
C20	0.0352 (8)	0.0463 (10)	0.0315 (8)	−0.0160 (7)	−0.0075 (6)	−0.0078 (7)
C21	0.0232 (6)	0.0421 (9)	0.0253 (7)	−0.0041 (6)	−0.0067 (5)	−0.0032 (6)
C22	0.0206 (5)	0.0266 (7)	0.0222 (6)	0.0001 (5)	−0.0031 (5)	−0.0035 (5)

### Geometric parameters (Å, º)

**Table:**

Br1A—C4	1.9064 (11)	C11—H11B	0.9800
Br1B—C4	1.9064 (15)	C11—H11C	0.9800
O1—C7	1.4406 (17)	C12—H12A	0.9800
O1—N1	1.4540 (14)	C12—H12B	0.9800
N1—C13	1.4922 (16)	C12—H12C	0.9800
N1—C9	1.5061 (18)	C13—C17	1.5274 (18)
C1—C2	1.3916 (19)	C13—C14	1.5409 (19)
C1—C6	1.3948 (19)	C13—H13A	1.0000
C1—C7	1.5165 (18)	C14—C15	1.5285 (19)
C2—C3	1.386 (2)	C14—C16	1.5350 (19)
C2—H2A	0.9500	C14—H14A	1.0000
C3—C4	1.389 (2)	C15—H15A	0.9800
C3—H3A	0.9500	C15—H15B	0.9800
C4—C5	1.376 (2)	C15—H15C	0.9800
C5—C6	1.3942 (19)	C16—H16A	0.9800
C5—H5A	0.9500	C16—H16B	0.9800
C6—H6A	0.9500	C16—H16C	0.9800
C7—C8	1.512 (2)	C17—C18	1.391 (2)
C7—H7A	1.0000	C17—C22	1.3975 (19)
C8—H8A	0.9800	C18—C19	1.396 (2)
C8—H8B	0.9800	C18—H18A	0.9500
C8—H8C	0.9800	C19—C20	1.383 (3)
C9—C10	1.535 (2)	C19—H19A	0.9500
C9—C11	1.536 (2)	C20—C21	1.384 (3)
C9—C12	1.536 (2)	C20—H20A	0.9500
C10—H10A	0.9800	C21—C22	1.392 (2)
C10—H10B	0.9800	C21—H21A	0.9500
C10—H10C	0.9800	C22—H22A	0.9500
C11—H11A	0.9800		
			
C7—O1—N1	111.94 (9)	C9—C11—H11C	109.5
O1—N1—C13	107.20 (9)	H11A—C11—H11C	109.5
O1—N1—C9	107.14 (9)	H11B—C11—H11C	109.5
C13—N1—C9	116.38 (11)	C9—C12—H12A	109.5
C2—C1—C6	118.76 (12)	C9—C12—H12B	109.5
C2—C1—C7	119.47 (12)	H12A—C12—H12B	109.5
C6—C1—C7	121.66 (12)	C9—C12—H12C	109.5
C3—C2—C1	121.23 (14)	H12A—C12—H12C	109.5
C3—C2—H2A	119.4	H12B—C12—H12C	109.5
C1—C2—H2A	119.4	N1—C13—C17	117.31 (10)
C2—C3—C4	118.61 (13)	N1—C13—C14	110.32 (11)
C2—C3—H3A	120.7	C17—C13—C14	112.06 (10)
C4—C3—H3A	120.7	N1—C13—H13A	105.4
C5—C4—C3	121.74 (11)	C17—C13—H13A	105.4
C5—C4—Br1A	120.07 (11)	C14—C13—H13A	105.4
C3—C4—Br1A	118.19 (11)	C15—C14—C16	108.65 (11)
C5—C4—Br1B	114.39 (18)	C15—C14—C13	110.80 (11)
C3—C4—Br1B	123.43 (18)	C16—C14—C13	111.31 (12)
Br1A—C4—Br1B	8.81 (16)	C15—C14—H14A	108.7
C4—C5—C6	118.94 (13)	C16—C14—H14A	108.7
C4—C5—H5A	120.5	C13—C14—H14A	108.7
C6—C5—H5A	120.5	C14—C15—H15A	109.5
C5—C6—C1	120.72 (13)	C14—C15—H15B	109.5
C5—C6—H6A	119.6	H15A—C15—H15B	109.5
C1—C6—H6A	119.6	C14—C15—H15C	109.5
O1—C7—C8	113.22 (12)	H15A—C15—H15C	109.5
O1—C7—C1	106.94 (10)	H15B—C15—H15C	109.5
C8—C7—C1	109.28 (12)	C14—C16—H16A	109.5
O1—C7—H7A	109.1	C14—C16—H16B	109.5
C8—C7—H7A	109.1	H16A—C16—H16B	109.5
C1—C7—H7A	109.1	C14—C16—H16C	109.5
C7—C8—H8A	109.5	H16A—C16—H16C	109.5
C7—C8—H8B	109.5	H16B—C16—H16C	109.5
H8A—C8—H8B	109.5	C18—C17—C22	118.35 (13)
C7—C8—H8C	109.5	C18—C17—C13	119.09 (12)
H8A—C8—H8C	109.5	C22—C17—C13	122.51 (12)
H8B—C8—H8C	109.5	C17—C18—C19	121.19 (15)
N1—C9—C10	107.73 (12)	C17—C18—H18A	119.4
N1—C9—C11	107.59 (11)	C19—C18—H18A	119.4
C10—C9—C11	108.01 (12)	C20—C19—C18	119.81 (16)
N1—C9—C12	115.25 (11)	C20—C19—H19A	120.1
C10—C9—C12	107.66 (11)	C18—C19—H19A	120.1
C11—C9—C12	110.36 (13)	C19—C20—C21	119.62 (15)
C9—C10—H10A	109.5	C19—C20—H20A	120.2
C9—C10—H10B	109.5	C21—C20—H20A	120.2
H10A—C10—H10B	109.5	C20—C21—C22	120.69 (15)
C9—C10—H10C	109.5	C20—C21—H21A	119.7
H10A—C10—H10C	109.5	C22—C21—H21A	119.7
H10B—C10—H10C	109.5	C21—C22—C17	120.29 (15)
C9—C11—H11A	109.5	C21—C22—H22A	119.9
C9—C11—H11B	109.5	C17—C22—H22A	119.9
H11A—C11—H11B	109.5		
			
C7—O1—N1—C13	−130.22 (11)	O1—N1—C9—C12	50.03 (14)
C7—O1—N1—C9	104.18 (12)	C13—N1—C9—C12	−69.86 (15)
C6—C1—C2—C3	−1.2 (2)	O1—N1—C13—C17	−58.63 (14)
C7—C1—C2—C3	175.04 (14)	C9—N1—C13—C17	61.23 (15)
C1—C2—C3—C4	0.7 (2)	O1—N1—C13—C14	71.30 (12)
C2—C3—C4—C5	0.1 (2)	C9—N1—C13—C14	−168.84 (10)
C2—C3—C4—Br1A	179.95 (11)	N1—C13—C14—C15	61.24 (14)
C2—C3—C4—Br1B	−171.8 (2)	C17—C13—C14—C15	−166.09 (11)
C3—C4—C5—C6	−0.3 (2)	N1—C13—C14—C16	−177.74 (11)
Br1A—C4—C5—C6	179.81 (11)	C17—C13—C14—C16	−45.06 (15)
Br1B—C4—C5—C6	172.24 (17)	N1—C13—C17—C18	−120.29 (14)
C4—C5—C6—C1	−0.2 (2)	C14—C13—C17—C18	110.60 (14)
C2—C1—C6—C5	0.9 (2)	N1—C13—C17—C22	62.25 (17)
C7—C1—C6—C5	−175.21 (14)	C14—C13—C17—C22	−66.86 (15)
N1—O1—C7—C8	95.05 (14)	C22—C17—C18—C19	−1.9 (2)
N1—O1—C7—C1	−144.54 (10)	C13—C17—C18—C19	−179.49 (14)
C2—C1—C7—O1	162.77 (13)	C17—C18—C19—C20	0.2 (2)
C6—C1—C7—O1	−21.13 (18)	C18—C19—C20—C21	1.1 (3)
C2—C1—C7—C8	−74.34 (17)	C19—C20—C21—C22	−0.7 (2)
C6—C1—C7—C8	101.77 (16)	C20—C21—C22—C17	−1.0 (2)
O1—N1—C9—C10	−70.17 (13)	C18—C17—C22—C21	2.3 (2)
C13—N1—C9—C10	169.94 (11)	C13—C17—C22—C21	179.79 (12)
O1—N1—C9—C11	173.61 (11)	C8—C7—C13—C14	4.56 (11)
C13—N1—C9—C11	53.72 (15)	C8—C7—N1—C9	172.28 (12)

### Hydrogen-bond geometry (Å, º)

**Table:**

*D*—H···*A*	*D*—H	H···*A*	*D*···*A*	*D*—H···*A*
C11—H11*B*···Br1*A*^i^	0.98	3.12	3.9782 (16)	148
C13—H13*A*···Br1*A*^ii^	1.00	3.07	4.0358 (13)	163
C13—H13*A*···Br1*B*^ii^	1.00	3.02	3.970 (2)	159
C15—H15*B*···Br1*B*^ii^	0.98	3.14	4.017 (3)	149

Symmetry codes: (i) −*x*, −*y*+1, −*z*+1; (ii) *x*+1, *y*−1, *z*.
